# Clinicopathological Determinants of Recurrence Risk and Survival in Mucinous Ovarian Carcinoma

**DOI:** 10.3390/cancers13225839

**Published:** 2021-11-21

**Authors:** Robert L. Hollis, Lorna J. Stillie, Samantha Hopkins, Clare Bartos, Michael Churchman, Tzyvia Rye, Fiona Nussey, Scott Fegan, Rachel Nirsimloo, Gareth J. Inman, C. Simon Herrington, Charlie Gourley

**Affiliations:** 1 The Nicola Murray Centre for Ovarian Cancer Research, Cancer Research UK Edinburgh Centre, MRC Institute of Genetics and Cancer, University of Edinburgh, Edinburgh EH4 2XU, Scotland, UK; l.j.j.stillie@sms.ed.ac.uk (L.J.S.); clare.bartos@ed.ac.uk (C.B.); mike.churchman@ed.ac.uk (M.C.); abbaema@rocketmail.com (T.R.); simon.herrington@ed.ac.uk (C.S.H.); charlie.gourley@ed.ac.uk (C.G.); 2 Cancer Research UK Beatson Institute, Institute of Cancer Sciences, University of Glasgow, Glasgow G61 1BD, Scotland, UK; gareth.inman@glasgow.ac.uk; 3 Edinburgh Cancer Centre, Western General Hospital, NHS Lothian, Edinburgh EH4 2LF, Scotland, UK; samantha.hopkins@nhslothian.scot.nhs.uk (S.H.); fiona.nussey@nhslothian.scot.nhs.uk (F.N.); rachel.nirsimloo@nhslothian.scot.nhs.uk (R.N.); 4 The Simpson Centre for Reproductive Health, Royal Infirmary of Edinburgh, NHS Lothian, Edinburgh EH16 4TJ, Scotland, UK; scott.fegan@nhslothian.scot.nhs.uk

**Keywords:** ovarian cancer, mucinous, survival, prognosis, relapse

## Abstract

**Simple Summary:**

Mucinous ovarian carcinoma (MOC) is a unique type of ovarian cancer. While many MOC patients have excellent survival, patients who experience recurrence have extremely poor prognosis. Identifying patients at the highest risk of recurrence is important for identifying which patients need the most aggressive treatment, and to identify where new treatment strategies are needed to improve survival. We use a large cohort of MOC patients to identify factors associated with high and low risk of recurrence. We show that once patients reach 5 years from diagnosis, their risk of recurrence is low. Patients with more advanced-stage disease and higher pathological grade of disease are more likely to experience recurrence, and their survival is significantly shorter. For early-stage MOC patients, survival time was similar whether they were treated with surgery plus chemotherapy, or whether they only had surgery. Patient survival time following recurrence is extremely poor (median 5 months); new treatment options are urgently needed to improve their survival.

**Abstract:**

Mucinous ovarian carcinoma (MOC) is a unique form of ovarian cancer. MOC typically presents at early stage but demonstrates intrinsic chemoresistance; treatment of advanced-stage and relapsed disease is therefore challenging. We harness a large retrospective MOC cohort to identify factors associated with recurrence risk and survival. A total of 151 MOC patients were included. The 5 year disease-specific survival (DSS) was 84.5%. Risk of subsequent recurrence after a disease-free period of 2 and 5 years was low (8.3% and 5.6% over the next 10 years). The majority of cases were FIGO stage I (35.6% IA, 43.0% IC). Multivariable analysis identified stage and pathological grade as independently associated with DSS (*p* < 0.001 and *p* < 0.001). Grade 1 stage I patients represented the majority of cases (53.0%) and demonstrated exceptional survival (10 year DSS 95.3%); survival was comparable between grade I stage IA and stage IC patients, and between grade I stage IC patients who did and did not receive adjuvant chemotherapy. At 5 years following diagnosis, the proportion of grade 1, 2 and 3 patients remaining disease free was 89.5%, 74.9% and 41.7%; the corresponding proportions for FIGO stage I, II and III/IV patients were 91.1%, 76.7% and 19.8%. Median post-relapse survival was 5.0 months. Most MOC patients present with low-grade early-stage disease and are at low risk of recurrence. New treatment options are urgently needed to improve survival following relapse, which is associated with extremely poor prognosis.

## 1. Introduction

Ovarian cancer remains a major cause of female cancer death, accounting for approximately 185,000 deaths globally per annum [1]. Mucinous ovarian carcinoma (MOC) is a distinct and uncommon ovarian cancer type [2,3,4]. MOC was previously thought to represent a greater proportion of ovarian carcinoma (OC) diagnoses (≥10%) [5]; however, a notable proportion of historic cases are now known to represent metastases from extra-ovarian sites, most commonly from the gastrointestinal tract [6,7]. True primary MOC is now recognized as an uncommon ovarian cancer type [2,6], representing ≤5% of ovarian carcinoma diagnoses [8].

In contrast to high-grade serous ovarian carcinoma (HGSOC), MOC is most frequently diagnosed at early clinical stage (mostly at FIGO stage I). These cases experience excellent long-term clinical outcome following surgical resection [3,9] and many do not routinely undergo systemic adjuvant chemotherapy [10,11]. However, unlike HGSOC, MOC demonstrates high levels of intrinsic chemoresistance [12]; while there is a paucity of data regarding response of MOC to first-line platinum-based chemotherapy, retrospective analyses in small advanced-stage MOC cohorts have suggested a response rate of 26–42% [13,14,15]. The GOG241 international phase III trial sought to compare the use of standard chemotherapy (carboplatin-paclitaxel) with oxaliplatin-capecitabine, carboplatin-paclitaxel-bevacizumab and oxaliplatin-capecitabine-bevacizumab for FIGO stage II–IV MOC or recurrent MOC [16]. Investigators reported response rates of 22%, 27%, 43% and 40% in these regimens, respectively, though the trial was terminated early due to slow recruitment. Advanced-stage MOC and recurrent MOC therefore represent a major clinical challenge, with traditional cytotoxic chemotherapy regimens generally proving ineffective in this context. Indeed, advanced-stage MOC—alongside advanced-stage clear cell ovarian carcinoma, which also demonstrates intrinsic chemoresistance—has been identified as an area of critical unmet clinical need [9].

In stark contrast to the excellent survival displayed by most MOC cases, the dismal prognosis reported in patients that experience relapse—alongside their poor response rate to chemotherapy—highlights the crucial need for detailed understanding of factors that contribute to recurrence risk. Better understanding of such factors is essential for improved patient prognostication and management, and for identification of MOC at greatest risk of relapse. Moreover, identified high-risk cases represent patients who may benefit most from inclusion in clinical trials of new therapeutic approaches.

Here, we seek to perform detailed characterization of the clinical landscape in MOC; we identify a contemporary real-world MOC patient cohort, investigate clinical determinants of survival outcome and identify factors associated with greatest risk of disease recurrence.

## 2. Materials and Methods

### 2.1. Cohort Identification

MOC patients diagnosed up to 31st December 2020 were identified using the Edinburgh Ovarian Cancer Database [9], wherein the diagnostic, treatment and outcome details of all ovarian cancer patients treated at the Edinburgh Cancer Centre are entered prospectively as part of routine clinical management. This study was conducted in accordance with the Declaration of Helsinki and was approved by the South East Scotland Cancer Information Research Governance Committee (Caldicott guardian approval CG/DF/E164, study reference CIR21021). For all subjects, informed consent was obtained or was waived by the ethics committee due to the retrospective nature of this study.

**Figure 1:**
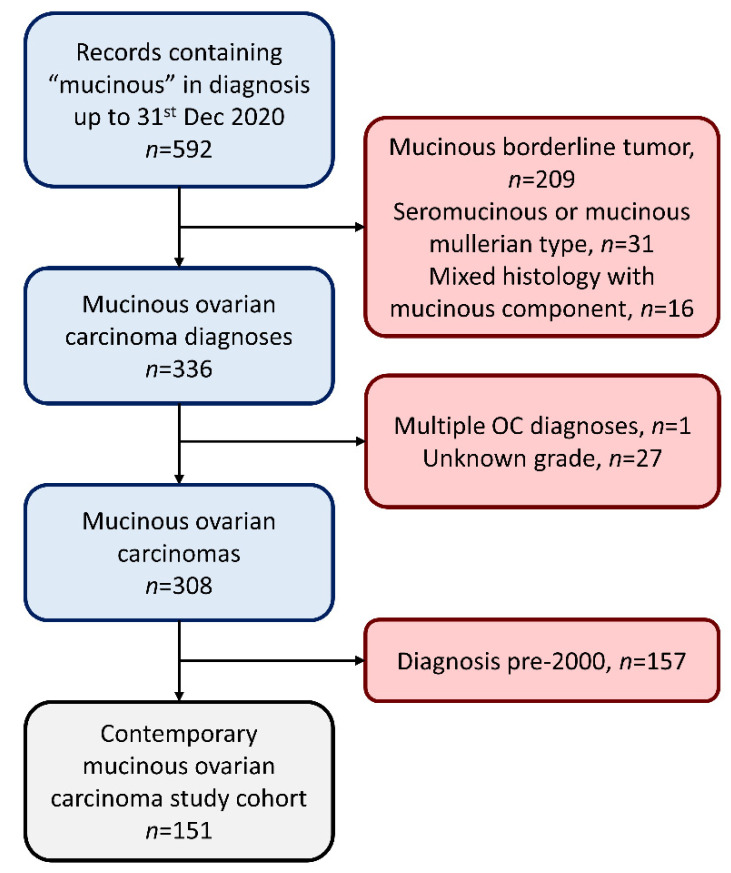
Identification of mucinous ovarian carcinoma patient cohort.

A total of 592 records were returned using the search term “mucinous” in the documented diagnostic histology (Figure 1). A total of 209 mucinous borderline tumors, 31 seromucinous/mucinous Mullerian-type tumors and 16 mixed histology mucinous-containing tumors were excluded, yielding 336 MOC diagnoses (Figure 1). One patient with multiple primary ovarian cancer diagnoses was excluded. A further 27 patients had MOC of unknown pathological grade and were excluded. Of the remaining 308 MOC, 157 cases were diagnosed prior to 1st January 2000, leaving 151 cases in the contemporary study cohort (Figure 1).

### 2.2. Survival Analysis

All statistical analyses were performed using R version 4.0.3 (R Foundation for Statistical Computing, Vienna, Austria). Survival analysis was performed within the Survival package [17] using Cox proportional hazards regression models and visualized using Kaplan–Meier plots. Overall and disease-specific survival (OS and DSS) were calculated from date of pathologically confirmed diagnosis. Progression-free survival (PFS) was calculated from date of pathologically confirmed diagnosis to date of disease recurrence/progression as determined by radiology or tumor marker. Progression dates determined by the treating physician were used in the absence of radiological/tumor marker investigations. Post-relapse survival was calculated as time from progression to patient death. Survival associations are presented as hazard ratios (HR) with corresponding 95% confidence intervals (95% CI). Median follow-up time was calculated using the reverse Kaplan–Meier method.

### 2.3. Additional Statistical Analysis

Categorical variables were compared using the Chi-squared or Fisher’s exact test, as appropriate. Continuous data were compared using the Mann–Whitney U test and visualized as violin plots. Relative risk of relapse according to clinicopathological features at diagnosis was quantified by risk ratio (RR) via comparison of relapsed MOC cases versus the relapse-free population.

## 3. Results

### 3.1. Cohort Characteristics

A total of 151 MOC patients diagnosed between 1 January 2000 and 31 December 2020 were identified (Figure 1). The median follow-up time was 5.7 years. A total of 27 patients experienced disease relapse/progression and 124 were relapse free at last follow-up. The 5 and 10 year DSS were 85.1% (95% CI 79.3–91.4%) and 82.6% (95% CI 76.1–89.7%). The 5 and 10 year PFS were 82.8% (95% CI 76.7–89.3%) and 78.1% (95% CI 70.6–86.5%).

The majority of cases presented with stage I disease (53 FIGO IA, 35.6% of evaluable cases and 64 FIGO IC, 43.0% of evaluable cases); 18 cases (12.1%) were FIGO stage II (1 IIA, 6 IIB and 11 IIC); 12 cases (8.1%) were FIGO stage III and 2 cases (1.3%) were FIGO stage IV. A total of 97 cases (64.2%) were grade 1 (well differentiated), 44 (29.1%) were grade 2 (moderately differentiated) and 10 (6.6%) were grade 3 (poorly differentiated).

Grade 1 and grade 2 cases demonstrated similar age at diagnosis (both median 52 years). The median age at diagnosis for grade 3 cases was 63 years; however, this was not significantly different compared to grade 1/2 cases (*p* = 0.210) (Figure S1).

### 3.2. First-Line Treatment

A total of 149 cases (98.7%) underwent primary debulking surgery (PDS) (Table 1); 90 did not undergo systemic adjuvant therapy following PDS and 59 underwent adjuvant chemotherapy after PDS (27 with single-agent carboplatin, 27 with carboplatin-paclitaxel combination, 1 with single-agent capecitabine, 4 with other platinum-containing regimens). A total of 5 of 53 stage IA patients underwent systemic adjuvant chemotherapy (4 with single-agent carboplatin, 1 with carboplatin-paclitaxel combination); 30 of 64 stage IC patients underwent adjuvant systemic chemotherapy (20 with single-agent carboplatin, 9 with carboplatin-paclitaxel combination, 1 with capecitabine).

### 3.3. Likelihood of Relapse following 2 and 5 Year Disease-Free Milestones

A total of 110 and 81 MOC patients were alive and disease free with subsequent follow-up at 2 and 5 years after diagnosis. From these landmark time points, the risk of subsequent relapse over the next 10 years was 8.3% (95% CI 1.2–14.9%) and 5.6% (95% CI 0.0–11.7%), respectively (Figure S2).

### 3.4. Clinical Determinants of Survival Outcome

**Figure 2:**
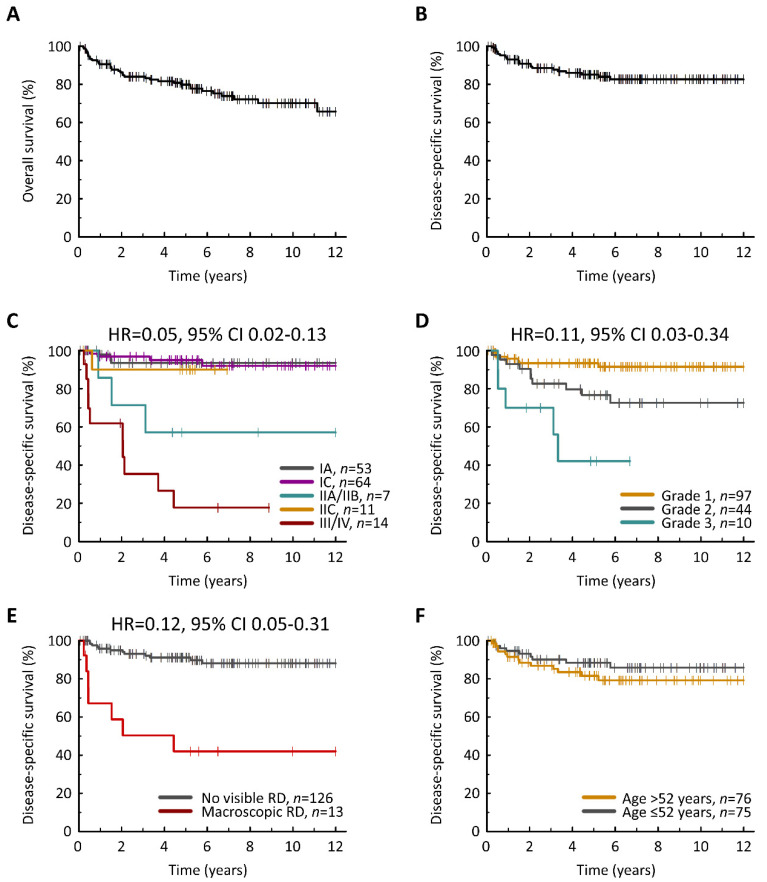
Clinical outcome in mucinous ovarian carcinoma. (**A**) Overall survival. (**B**) Disease-specific survival (DSS). (**C**) DSS according to FIGO stage at diagnosis; labelled hazard ratio (HR) represent comparison of stage I vs. III/IV. (**D**) DSS according to disease grade; labelled HR represents comparison of grade 1 vs. grade 3 cases. (**E**) DSS according to residual disease (RD) status following surgical debulking. (**F**) DSS according to age at diagnosis. HR for age as a continuous variable = 1.03 (95% CI 1.00–1.06).

Univariable DSS analysis identified FIGO stage at diagnosis (HR for FIGO I vs. III/IV = 0.05, 95% CI 0.02–0.13, *p* < 0.001) (Figure 2C), pathological disease grade (HR for grade 1 vs. 3 = 0.11, 95% CI 0.03–0.34, *p* < 0.001) (Figure 2D), residual disease (RD) status (HR for no visible RD vs. macroscopic RD = 0.12, 95% CI 0.05–0.31, *p* < 0.001) (Figure 2E) and age at diagnosis (HR = 1.03, 95% CI 1.00–1.06, *p* = 0.049) (Figure 2F) as significantly associated with survival (Table 2). Multivariable analysis demonstrated that only FIGO stage and disease grade were independently associated with DSS (*p* < 0.001 and *p* < 0.001). These data were mirrored upon analysis of PFS (Table 2, Figure S3).

### 3.5. Characteristics of Relapsed MOC

**Figure 3:**
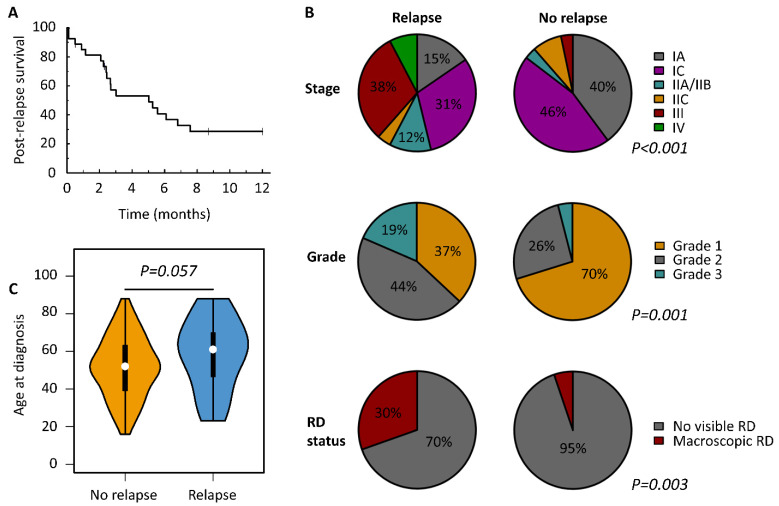
Characteristics of relapsed mucinous ovarian carcinoma. (**A**) Overall post-relapse survival. (**B**) Clinicopathological features at diagnosis in cases who subsequently experienced relapse versus those relapse free at last follow-up; (**C**) violin plot of age at diagnosis between relapsed cases and cases that that were relapse free at last follow-up.

The median time to relapse was 12.4 months (95% CI 7.9–21.4). Median post-relapse survival was 5.0 months (95% CI 2.4–16.3) (Figure 3A).

Grade 2 and 3 cases were significantly over-represented in the relapsed MOC cohort (12 and 5 cases, 44.4% and 18.5%) compared to the relapse-free population (32 and 5 cases, 25.8% and 4.0%) with corresponding depletion of grade 1 cases (10/27, 37.0% vs. 87/124, 70.2%) (*p* = 0.001) (Figure 3B, Table 3). The risk ratio (RR) for relapse in grade 1 and 2 cases compared to grade 3 MOC was 0.21 (95% CI 0.09–0.48) and 0.55 (95% CI 0.25–1.20). The likelihood of grade 1, 2 and 3 MOC patients remaining disease free 5 years following diagnosis was 89.5%, 74.9% and 41.7% (Figure S3C).

Patients presenting with FIGO stage I disease, and completely debulked patients (no visible RD) were under-represented in relapsed cases compared to the relapse-free population (*p* < 0.001 and *p* = 0.003) (Figure 3B, Table 3). The RR for relapse in stage I and stage II cases versus stage III/IV was 0.14 (95% CI 0.08–0.27) and 0.31 (95% CI 0.12–0.79). The RR for no visible RD was 0.24 (95% CI 0.12–0.47). The likelihood of FIGO stage I, II and III/IV MOC patients remaining disease free 5 years following diagnosis was 91.1%, 76.7% and 19.8% (Figure S3B).

The relapsed population displayed a higher age at diagnosis compared to the relapse-free population (median 61 vs. 52 years), but this did not reach statistical significance (*p* = 0.057) (Figure 3C).

### 3.6. Outcome in Low-Risk Mucinous Ovarian Carcinoma

FIGO stage I grade 1 MOC represent a large proportion of MOC diagnoses (53.0%, 79 of 149 evaluable cases in our cohort) and are widely considered a markedly low-risk patient population. The 10 year DSS and PFS within the FIGO I grade 1 MOC population was 95.6% (95% CI 90.9–100.0%) and 89.3% (95% CI 80.7–98.7%) (Figure S4A,B). Within this population, stage IA and stage IC cases demonstrated similar survival (Figure S4C,D). Those who received adjuvant chemotherapy demonstrated similar outcome to those treated with surgery alone (Figure S4E,F); this remained consistent when considering only stage IC cases, who are typically recommended adjuvant chemotherapy (Figure S4G,H).

## 4. Discussion

MOC is a unique type of ovarian cancer. Detailed understanding of clinicopathological features associated with outcome and relapse risk is crucial for improving patient prognostication, management, and identification of patients at high risk of disease recurrence who should be enrolled in clinical trials of new treatment strategies. Here, we report one of the largest MOC patient cohorts to date, identifying clinicopathological features associated with recurrence risk and survival.

We observe excellent survival across the overall study population (5 year DSS 85.1%, 5 year PFS 82.8%), consistent with previous reports of high survival rates in MOC [3,18,19]. A recent Surveillance, Epidemiology and End Results (SEER) analysis of MOC reported a 5 year OS 82.9% in localized disease [3]. Similarly, Mueller et al. reported 80% PFS in stage I/II MOC [19]. We report a 5 year DSS of 17.7% in stage III/IV MOC, similar to the 13.9% reported for distantly disseminated disease in the recent SEER analysis. This is consistent with both the short PFS and OS time reported by Hess et al. in stage III/IV MOC (median 5.7 and 12.0 months) [13] and the identification of MOC as the highest risk histological type in stage IV ovarian carcinomas [20].

We did not observe apparent differences in outcome between grade I stage IA and IC cases, or between grade I stage IC cases who did or did not receive adjuvant chemotherapy; this is consistent with recent reports demonstrating no survival benefit for stage IC MOC patients undergoing adjuvant chemotherapy [21]. Substaging information was unavailable for the majority of our stage IC cases, precluding the ability to perform substage-specific comparisons. Future investigations should seek to investigate the impact of disease substages, in particular of surgical spill (stage IC1) versus stage IA patients.

Fewer studies have investigated whether pathological grade provides meaningful additional prognostic information independent of stage. Within our cohort, 64%, 29% and 7% of cases were grade 1, 2 and 3, respectively. We demonstrate a significant association between pathological grade and survival, independent of other clinicopathological factors including tumor stage. These data are in agreement with pan-histotype analyses in large ovarian cancer cohorts [18].

The likelihood of subsequent relapse after reaching specific disease-free milestones is of great clinical interest to both patients and clinicians, particularly with regard to time points where follow-up frequency is reduced, or where patients are discharged for follow-up with their primary care practitioner. We show that across our MOC cohort, the risk of relapse after reaching 2 and 5 year relapse-free milestones is low (approximately 8% and 6%, respectively). This is in line with other reports suggesting late relapse is uncommon in MOC [9,19]; however, investigators have not typically sought to quantify subsequent risk at specific disease-free time points. While relapse risk is known to reduce over time, quantification of patient risk at specific time points is clinically valuable.

A number of previous reports have associated clinical features with PFS time [3,13,19]; however, there has been limited description of relapsed cases and comparison against relapse-free MOC to identify clinicopathological features over- and under- represented at relapse. We quantify recurrence risk factors by both PFS analysis and direct comparison of relapsed and relapse-free populations. MOC patients diagnosed at early stage are at significantly reduced risk of relapse compared to advanced-stage cases. Low-grade MOC are also at reduced risk. While presence of RD was a major risk factor for recurrence, multivariable analysis demonstrated that its association with PFS and DSS was not independent of other factors. This is likely due to relationship between RD status and stage at diagnosis.

The relapsed MOC population demonstrated evidence for older age at diagnosis compared to the relapse-free population, though this did not cross the threshold for statistical significance (*p* = 0.057). Univariable analysis identified age at diagnosis as significantly associated with shorter DSS and PFS. However, there was no significant association after adjusting for other clinicopathological factors. These findings may be explained by the older age at diagnosis we observed in grade 3 MOC (median 63 years), which itself is significantly associated with increased recurrence risk and shorter survival time.

We demonstrate that recurrent MOC is associated with dismal prognosis: the median post-relapse survival time in our relapsed MOC population was just 5.0 months, similar to the outcome of advanced-stage colorectal cancer patients [22]. This is considerably shorter than post-relapse survival time reported in platinum-sensitive or platinum-resistant HGSOC, or in endometrioid OC [23,24]. Low-grade serous ovarian carcinoma (LGSOC)—more similar to MOC in its inherent chemoresistance and low prevalence (5% cases)—also demonstrates markedly longer post-relapse survival time compared to MOC [25]. These data highlight the urgent need for new treatment options to improve the survival of relapsed MOC patients.

However, the rarity of true MOC represents a significant obstacle toward performing disease-specific trials of new treatment regimens. Indeed, the phase III GOG241 trial of stage II–IV or recurrent MOC demonstrated poor recruitment [16], despite recruiting internationally between the US and UK, ultimately contributing to early trial termination. Inclusion of MOC in modern BASKET trials of molecular agents therefore represents the most promising avenue by which to discover novel agents with the potential to improve the survival of advanced-stage and relapsed MOC patients [26]. Ongoing studies involving MOC patients include investigations of immunotherapies (e.g., NCT02839707 and NCT04739800), anti-angiogenic agents (e.g., NCT01081262 and NCT02923739) and inhibition of WEE1 (NCT02101775).

While clinicopathological factors have a large impact on patient risk, a minority of cases in poor prognosis groups demonstrate long-term survival; similarly, a proportion of patients with early-stage and/or low-grade disease experience recurrence. Molecular characterization of relapsed and relapse-free MOC cohorts has the potential to identify both biomarkers of recurrence risk and molecular targets for biological agents in MOC.

A major strength of this study is the large numbers of an uncommon tumor type from a single center with detailed, prospectively collected clinical annotation. Confinement of the cohort to exclude historic (pre-2000) cases known to harbor a significant number of extra-ovarian metastases is also a major strength. However, the lack of contemporary pathological review is a significant limitation; while our cohort represents a contemporary MOC population, the distinction between true primary MOC and extra-ovarian metastases remains a significant challenge in modern gynecological pathology. IHC for combinations of CK7, CK20, CEA, CDX2, CA125 and PAX8 were used to aid diagnosis in the majority of recently diagnosed cases, improving the fidelity of diagnosis [27]. However, we cannot exclude the possibility that our study population contains a minority of extra-ovarian cases masquerading as true primary MOC.

The limited number of advanced-stage MOC cases, due to the relative rarity of advanced stage at diagnosis in MOC, represents a further weakness of this study. A subgroup analysis specifically of advanced-stage cases was therefore not possible. Moreover, treatment regimens were heterogeneous across the cohort, primarily due to differences in recommendations for first-line management of MOC according to disease stage and pathological grade. Evolving therapeutic approaches over time also account for part of the heterogeneity in treatment; this is a limitation of the extensive study period. However, due to the rarity of MOC, extensive study periods are required to identify enough cases for sufficiently powered analyses. Detailed assessment of relapsed cases and comparison against the relapse-free MOC population represents a further strength of this work versus previous investigations of MOC.

## 5. Conclusions

Patients diagnosed with MOC are likely to experience excellent long-term survival. Cases of grade 1 MOC presenting at FIGO stage I are a particularly low-risk population. Patients who are disease free 5 years after diagnosis are unlikely to subsequently relapse. Advanced stage at diagnosis and higher pathological grade are associated with poorer survival; these cases—alongside those with macroscopic RD following debulking surgery—are over-represented in patients who experience relapse. Post-relapse survival in recurrent MOC is extremely poor and further treatment options are urgently needed to improve clinical outcomes in this context.

## Figures and Tables

**Table 1:** Characteristics of mucinous ovarian carcinoma study cohort.

		N	%
**Total cases**	N	151	
**Age at diagnosis**	Median years	52	range 16–88
**Year of diagnosis**	2000–2004	33	21.9
	2005–2009	39	25.8
	2010–2014	45	29.8
	2015 onwards	34	22.5
**Pathological grade ^a^**	Grade 1	97 ^b^	64.2
	Grade 2	44	29.1
	Grade 3	10 ^c^	6.6
**FIGO stage at diagnosis ^d^**	IA	53	35.6
	IC	64 ^e^	43
	IIA	1	0.7
	IIB	6	4
	IIC	11	7.4
	III	12 ^f^	8.1
	IV	2	1.3
	NA	2	-
**RD following first-line debulking**	No visible RD	126	90.6
	Macroscopic RD	13 ^g^	9.4
	NA	12	-
**First-line treatment regime**	PDS only	90	59.6
	PDS + carboplatin	27	17.9
	PDS + carboplatin-paclitaxel	27	17.9
	PDS + other platinum-containing combination	4 ^h^	2.6
	PDS + capecitabine	1	0.7
	Neoadjuvant carboplatin-paclitaxel	1 ^i^	0.7
	None	1 ^j^	0.7
**Status at last follow-up**	No known active disease	124	82.1
	Relapsed/progressed	27	17.9
**Vital status at last follow-up**	Alive	115	76.2
	Deceased—died of OC	22	14.6
	Deceased—other causes	14	9.3
**Median follow-up time**	Years	5.73 ^k^	95% CI 5.21–6.78

^a^ By the International Federation of Gynecology and Obstetrics (FIGO) grading system following recommendation of this system in 2010. Grading methods were more heterogeneous for earlier diagnoses due to the lack of official guidelines for MOC grading. For diagnoses from 2009 onwards, MOC diagnoses were noted to include IHC for combinations of CK7, CK20, CEA, CDX2, ER, PAX8 and CA125 in 53/90 (59%) of cases. Prior to 2009, use of diagnostic IHC was not recorded; ^b^ includes 14 cases documented as low grade; ^c^ includes 1 case documented as high grade. A total of 4 were stage IA, 4 IC, 1 IIB, 1 IIIA; ^d^ according to contemporary International Federation of Gynecology and Obstetrics (FIGO) staging system at time of diagnosis; ^e^ 13 IC1, 7 IC2, 2 IC3, 42 IC not otherwise specified; ^f^ 3 IIIA, 8 IIIC, 1 stage III not otherwise specified: 5 achieved no RD, 6 had macroscopic RD, 1 has unknown RD status; ^g^ 2 cases with <2 cm RD, 8 cases with ≥2 cm RD, 1 case with macroscopic RD of unknown size, 2 cases who did not undergo first-line debulking surgery; ^h^ 1 with paclitaxel and bevacizumab, 1 with paclitaxel and gemcitabine, 2 with capecitabine and bevacizumab; ^i^ progression before interval debulking could be attempted; ^j^ biopsy only—unfit for treatment; ^k^ 17 cases (11.3%) alive and recurrence free with <3 years follow-up. NA, not available; FIGO, International Federation of Gynecology and Obstetrics; RD, residual disease; OC, ovarian carcinoma.

**Table 2:** Survival analysis of mucinous ovarian carcinoma patients.

		Disease-Specific Survival						Progression-Free Survival					
		Univariable Analysis			Multivariable Analysis			Univariable Analysis			Multivariable Analysis		
		HR	95% CI	*p*-Value	HR	95% CI	*p*-Value	HR	95% CI	*p*-Value	HR	95% CI	*p*-Value
**FIGO stage at diagnosis**	IA	0.05	0.01–0.18	<0.0001	0.02	0.00–0.18	<0.001	0.06	0.02–0.19	<0.0001	0.04	0.01–0.23	<0.001
	IC	0.05	0.02–0.15	<0.0001	0.07	0.02–0.30	<0.001	0.09	0.04–0.23	<0.0001	0.14	0.04–0.48	0.002
	IIA/B	0.37	0.10–1.35	0.132	0.33	0.07–1.50	0.150	0.36	0.10–1.31	0.121	0.37	0.09–1.62	0.187
	IIC	0.08	0.01–0.60	0.014	0.24	0.03–2.19	0.205	0.08	0.01–0.64	0.017	0.23	0.03–1.99	0.181
	III/IV	ref	ref	ref	ref	ref	ref	ref	ref	ref	ref	ref	ref
**Pathological grade**	Grade 1	0.11	0.03–0.34	<0.001	0.05	0.01–0.22	<0.001	0.14	0.05–0.41	<0.001	0.08	0.02–0.31	<0.001
	Grade 2	0.35	0.12–1.02	0.055	0.15	0.04–0.65	0.011	0.39	0.14–1.11	0.078	0.26	0.08–0.92	0.037
	Grade 3	ref	ref	ref	ref	ref	ref	ref	ref	ref	ref	ref	ref
**RD following first-line debulking**	No visible RD	0.12	0.05–0.31	<0.0001	0.41	0.10–1.76	0.232	0.16	0.06–0.38	<0.0001	0.60	0.17–2.09	0.418
	Macroscopic RD	ref	ref	ref	ref	ref	ref	ref	ref	ref	ref	ref	ref
**Age at diagnosis**	Years	1.03	1.00–1.06	0.037	1.00	0.96–1.04	0.899	1.03	1.00–1.05	0.033	1.02	0.99–1.05	0.217

HR, hazard ratio; CI, confidence interval; FIGO, International Federation of Gynecology and Obstetrics; ref, reference population; RD, residual disease.

**Table 3:** Characteristics of relapsed and relapse-free mucinous ovarian carcinoma.

		Relapsed/Progressed		Recurrence Free at Last Follow-Up		
		N	%	N	%	*p*-Value
**Cases**	N	27	-	124	-	-
**Age at diagnosis**	Median years	61	range 23–88	52	range 16–88	*p* = 0.057 ^+^
**Year of diagnosis**	2000–2004	4	14.8	29	23.4	*p* = 0.777 ^^^
	2005–2009	8	29.6	31	25	
	2010–2014	8	29.6	37	29.8	
	2015 onwards	7	25.9	27	21.8	
**Pathological grade**	Grade 1	10	37	87	70.2	*p* = 0.001 ^^^
	Grade 2	12	44.4	32	25.8	
	Grade 3	5	18.5	5	4	
**FIGO stage at diagnosis**	IA	4	15.4	49	39.8	*p* < 0.001 ^#^
	IC	8	30.8	56	45.5	
	IIA	0	0	1	0.8	
	IIB	3	11.5	3	2.4	
	IIC	1	3.8	10	8.1	
	III	8	30.8	4	3.3	
	IV	2	7.7	0	0	
	NA	1	-	1	-	
**RD following surgical debulking**	No visible RD	16	69.6	110	94.8	*p* = 0.003 ^~^
	Macroscopic RD	7	30.4	6	5.2	
	NA	4	-	8	-	
**Treatment at relapse**	Surgery	5 ^a^	18.5	-	-	-
	Surgery + platinum-containing regime	2 ^b^	7.4	-	-	
	Single-agent platinum	2	7.4	-	-	
	Platinum-taxane	2	7.4	-	-	
	Other cytotoxic regime	4 ^c^	14.8	-	-	
	Radiotherapy	1	3.7	-	-	
	Letrozole	1	3.7	-	-	
	Awaiting decision at last follow-up	1	3.7	-	-	
	No active therapy—palliation only	9	33.3	-	-	
**Time to relapse**	Median months	12.4	95% CI 7.9–21.4	-	-	-
**Post-relapse survival**	Median months	5.0	95% CI 2.4–16.3	-	-	-

^+^ Mann–Whitney U test; ^^^ Chi-squared test; ^#^ Chi-squared test FIGO I vs. FIGO II/III/IV; ^~^ Fisher’s exact test. ^a^ 1 with letrozole treatment; ^b^ 1 single-agent carboplatin, 1 oxaliplatin plus capecitabine; ^c^ 1 cisplatin plus etoposide, 1 carboplatin plus paclitaxel switched to cisplatin plus etoposide, 1 oxaliplatin plus capecitabine, 1 FOLFIRI (irinotecan plus leucovirin plus 5-fluorouracil), 1 paclitaxel plus Hedghehog pathway inhibitor (phase I trial). NA, not available; FIGO, International Federation of Gynecology and Obstetrics; RD, maximal residual disease diameter; OC, ovarian carcinoma.

## Data Availability

We are happy to provide summary data upon reasonable request. Per-patient level data cannot be provided in order to comply with our research ethics framework.

## Supplementary Materials

The following are available online at https://www.mdpi.com/article/10.3390/cancers13225839/s1, Figure S1: Violin plot of patient age at diagnosis by pathological grade at diagnosis, Figure S2: Risk of subsequent recurrence at specific disease-free milestones, Figure S3: Progression-free survival (PFS) of mucinous ovarian carcinomas, Figure S4: Clinical outcome in grade 1 FIGO stage I mucinous ovarian carcinoma.
