# Supplementary material for: Clinicopathological Determinants of Recurrence Risk and Survival in Mucinous Ovarian Carcinoma
Source: Cancers (Basel). 2021 Nov 21;13(22):5839. doi: 10.3390/cancers13225839 (PMC8616033; doi:10.3390/cancers13225839)
Supplement: Supplementary file 1 [file cancers-13-05839-s001.zip › cancers-1420098-supplementary.pdf]

Supplementary Materials:

Clinicopathological Determinants of Recurrence Risk and Survival in Mucinous Ovarian Carcinoma

Robert L. Hollis, Lorna J. Stillie, Samantha Hopkins, Clare Bartos, Michael Churchman, Tzyvia Rye, Fiona Nussey, Scott Fegan, Rachel Nirsimloo, Gareth J. Inman, C. Simon Herrington and Charlie Gourley

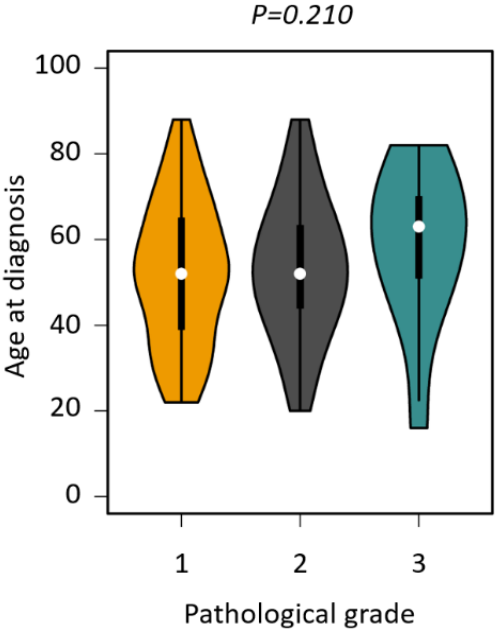

Figure S1. Violin plot of patient age at diagnosis by pathological grade at diagnosis. Labelled P-value represents comparison of grade 3 vs. grade 1/2 cases.

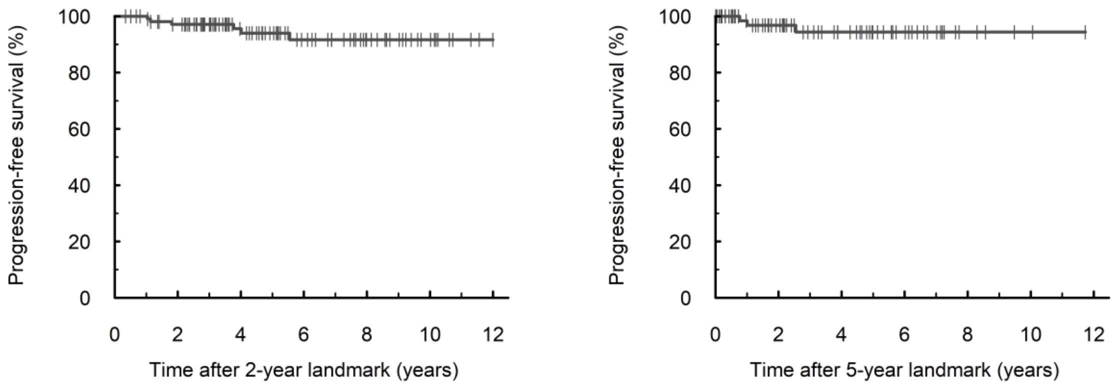

Figure S2. Risk of subsequent recurrence at specific disease-free milestones. (A) Progression-free survival (PFS) following 2 year disease-free interval. (B) PFS following 5 year disease-free interval.

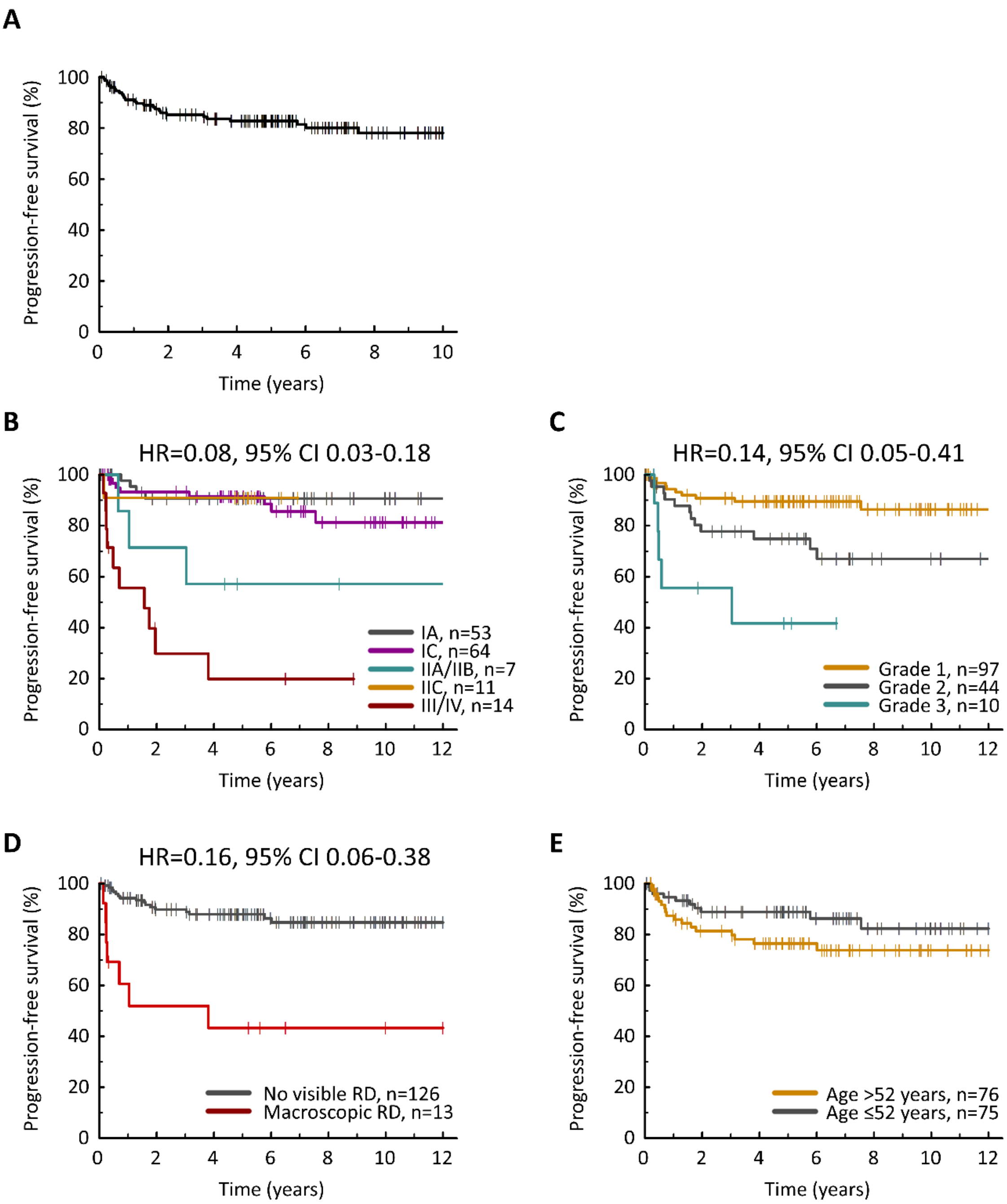

Figure S3. Progression-free survival (PFS) of mucinous ovarian carcinomas. (A) Overall PFS across the cohort. (B) PFS according to FIGO stage at diagnosis; labelled hazard ratio (HR) represents comparison of stage I versus stage III/IV. (C) PFS according to pathological grade at diagnosis; labelled HR represents comparison of grade 1 vs. grade 3 cases. (D) PFS according to residual disease (RD) status. (E) PFS according to age at diagnosis; HR for age as a continuous variable=1.03 (95% CI 1.00-1.05).

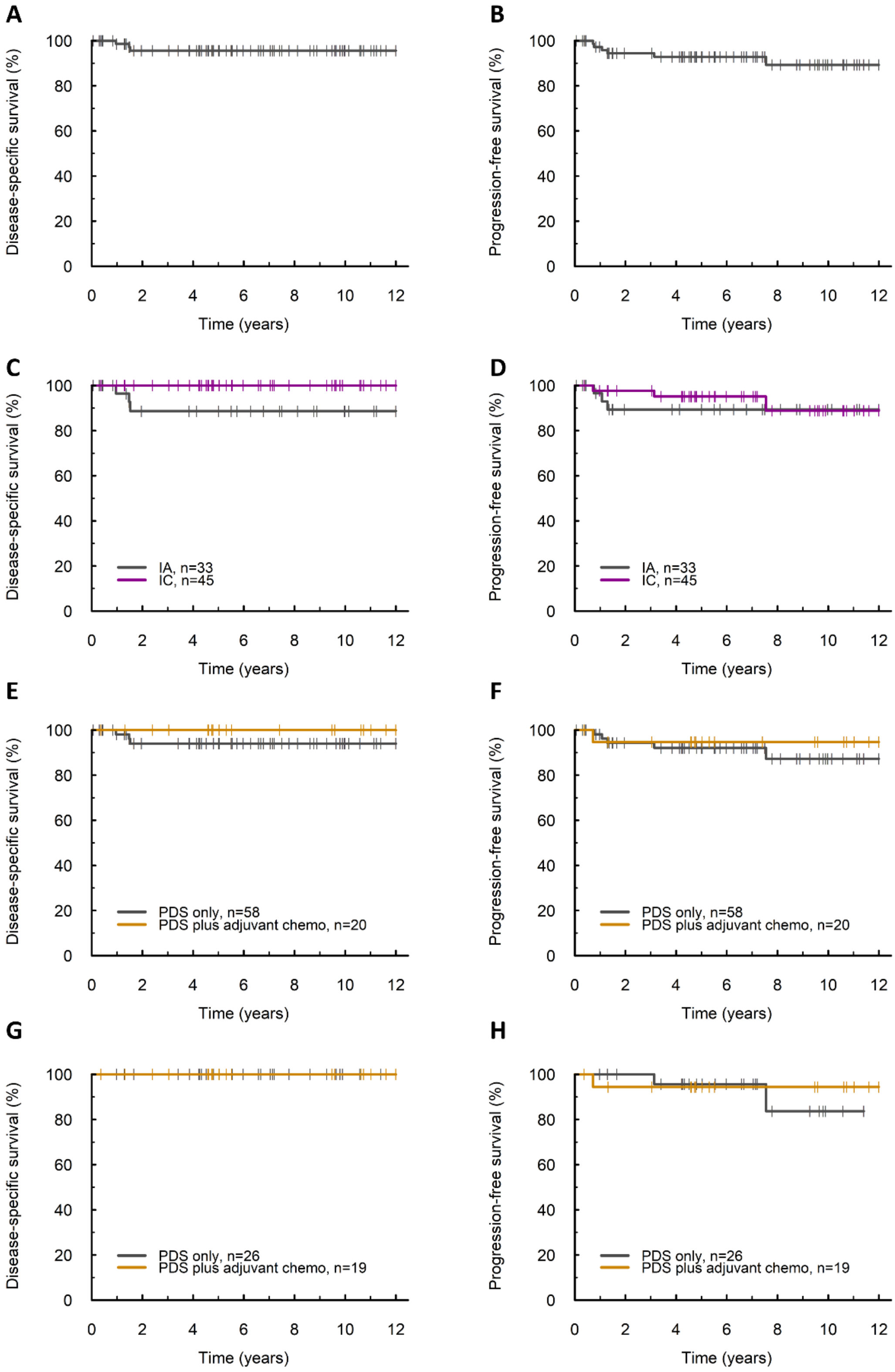

Figure S4. Clinical outcome in grade 1 FIGO stage I mucinous ovarian carcinoma. (A) Overall disease-specific survival (DSS). (B) Overall progression-free survival (PFS). (C) DSS according to FIGO stage at diagnosis. (D) PFS according to FIGO stage at diagnosis. (E) DSS according to first-line treatment regime. (F) PFS according to first-line treatment regime. (E) DSS according to first-line treatment regime in stage IC patients only. (F) PFS according to first-line treatment regime in stage IC patients only. PDS, primary debulking surgery.
